# An Adaptive Human Posture Detection Algorithm Based on Generative Adversarial Network

**DOI:** 10.1155/2022/7193234

**Published:** 2022-03-31

**Authors:** Zhiming Xu, Wenzheng Qu, Hanhua Cao, Meixia Dong, Danyu Li, Zemin Qiu

**Affiliations:** ^1^Guangzhou Xinhua University, Guangzhou 510000, Guangdong, China; ^2^University of Edinburgh, Edinburgh EH9 3JN, UK

## Abstract

Human posture equipment technology has advanced significantly thanks to advances in deep learning and machine vision. Even the most advanced models may not be able to predict all body joints accurately. This paper proposes an adaptive generative adversarial network to improve the human posture detection algorithm in order to address this issue. GAN is used in the algorithm to detect human posture improvement. The algorithm uses OpenPose to detect and connect keypoints and then generates heat maps in the GAN system model. During the training process, the confidence evaluation mechanism is added to the system model. The generator predicts posture, while the resolver refines human joints over time. And, by using normalization technologies in the confidence evaluation mechanism, the generator can pay more attention to the prominent body joints, improving the algorithm's body detection accuracy of nodes. In MPII, LSP, and FLIC datasets, the proposed algorithm has shown to have a good detection effect. Its positioning accuracy is about 95.37 percent, and it can accurately locate the joints of the entire body. Several other algorithms are outperformed by this one. The algorithm described in this article has the best simultaneous runtime in the LSP dataset.

## 1. Introduction

The goal of human posture estimation is to predict positions from the input images of body joints. At the moment, human posture estimation relies on a variety of methods [1–6], such as Google's G-RMI, which uses target detection to open a human posture estimation method. The OpenPose system [7–9] proposed by Carnegie Mellon University (CMU) greatly improved the construction of human posture. OpenPose is an open-source library written in the Caffe programming language that can track human facial expressions, torsos, limbs, and even fingers. It is based on convolutional neural networks and supervised learning, not only for a single person, but also for a group. The introduction of AlphaPose has significantly improved the accuracy of human pose detection. The AlphaPose system [10,11] is a highly accurate real-time multiplayer posture estimation system. The algorithm uses a top-down structure, that is, to detect the inverted human body first and then obtain the key points and skeleton. Therefore, his accuracy and Ap values are higher than OpenPose. But the disadvantage is that as the number of people on the picture increases, his calculations increase and the speed becomes slower. These algorithms still have the problem of being frustrated in the face of occlusion problems—problems such as high accuracy, obstruction of real time, and limited small size images.

This article proposes an adaptive generative adversarial network to improve the human pose detection algorithm in order to address the aforementioned issues. Based on the robustness of the generative adversarial network (GAN) in occlusion conditions, the coherence of the shape, and the fidelity of the details, the algorithm improves the generative adversarial network (GAN) model [12–16]. The generator generates the human body posture, the discriminator distinguishes the actual heat map, and the human joint is gradually improved. The existing posture resolution effect is improved through continued training. Finally, the normalization technology is used to perform confidence processing on the generator's generated data in order to keep the correct key nodes, improving the algorithm's body node detection accuracy. Through experiments, compared with the existing methods, the proposed algorithm can improve the accuracy of the position of known people and search for poses in these areas through the GAN model.

## 2. This Article Algorithm

Human posture estimation is a very basic problem in computer vision. From the perspective of the name, it can be understood as an estimate of the position of the posture of the “human body” (key points, such as head, left hand, right foot, etc.). The traditional human skeleton key point detection algorithm is basically based on the idea of template matching on the basis of geometric a priori, so the core lies in how to use the template to represent the entire human body structure, including the representation of key points, the representation of limb structure, and the representation of the relationship between different limb structures. A good template matching idea can simulate more attitude ranges, so that it can better match and detect the corresponding human posture.

Human posture estimation can be divided into two lines of thought:

1. “top-down,” which refers to the detection of human body areas first and then the detection of key points in the human body in the area.
2. “bottom-up:” which refers to detecting all the key points of the human body in the picture first and then corresponding these key points to different individual characters. It should be mentioned here that the first scheme is slower because it needs to detect each area of the human body separately for forward key point detection, while OpenPose uses the second scheme.

There are disadvantages of the “bottom-up” method: (1) the priori information of the global context is not used, that is, the key points of other people's bodies in the picture. (2) Corresponding key points to different individuals, the algorithm complexity is too high.

OpenPose improvement point: in the proposed “Part Affinity Fields (PAFs),” each pixel is a 2D vector for characterizing position and direction information. Based on the detected joint nodes and joint connection areas, using the greedy inference algorithm, these joint nodes can be quickly corresponded to different individual characters.

### 2.1. Heat Map

#### 2.1.1. Critical Point Detection

**Figure 1:**
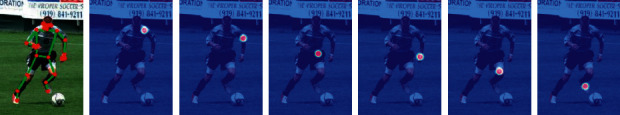
Key point probability distribution plot.

Calculate the ground truth of S through the 2D points *x*_*j*,*k*_ and *x*_*i*,*k*_ marked in the image. Among them, *x*_*j*_1_,*k*_ and *x*_*j*_2_,*k*_ represent the real pixels corresponding to the two key points *j*_1_ and *j*_2_ of a certain person *k* in Figure 1. Calculation method: when the pixel point *p* is close to the annotation points *x*_*j*,*k*_ and *x*_*i*,*k*_, it reaches the peak of the normal curve. Then the S of the *j-*th joint in each image is the peak of the normal distribution of *k* individuals in the image.

Figure 1 is the heat map of each joint point. The first picture on the left is the input image and the final predicted joint position. The second picture is the probability result predicted by the channel responsible for the neck node. Red and yellow represent the high probability that the corresponding pixel location is the neck. The other blue areas mean that it will hardly be the neck location. The following pictures, respectively, represent the *p* probability results of different nodes predicted by different channels of heat map.

(1) $$\begin{array}{c} S_{j,k}^{*}=\exp\left(-\frac{{\left\|p-x_{j,k}\right\|}_{2}^{2}}{\sigma^{2}}\right),\\ S_{j}^{*}\left(p\right)=\underset{k}{\min}S_{j,k}^{*}\left(p\right). \end{array}$$

#### 2.1.2. Joint Connections

If a pixel *p* is located on this torso, the value of *L*_*c*,*k*_^*∗*^(*p*) represents the unit vector from the key point *j*_1_ to the key point *j*_2_. For pixels not on the torso, the corresponding vector is the zero vector.

Then the value of *L*_*c*,*k*_^*∗*^(*p*) is as follows:

(2) $$\begin{array}{c} L_{c,k}^{*}\left(p\right)=\left\{\begin{array}{cc} v, & \text{if p on c},k,\\ 0, & \text{otherwise.} \end{array}\right) \end{array}$$

Among them, *v*=(*x*_*j*_2_,*k*_ − *x*_*j*_1_,*k*_)/|*x*_*j*_2_,*k*_ − *x*_*j*_1_,*k*_|_2_ represents the unit direction vector corresponding to this torso.

Pixels belonging to this torso satisfy the following function:

(3) $$\begin{array}{c} 0\leq v\left(p-x_{j_{1},k}\right)\leq l_{c,k},\\ \left|v_{⊥}\left(p-x_{j_{1},k}\right)\right|\leq \sigma_{l}. \end{array}$$

The inner table *σ*_*l*_ shows the distance between pixels. The length of the torso is *l*_*c*,*k*_=|*x*_*j*_2_,*k*_ − *x*_*j*_1_,*k*_|_2_, *v*_⊥_ represents the vector perpendicular to *v*. The whole image is the average value corresponding to all people in the image. *n*_*c*_(*p*) is the number of nonzero vectors corresponding to *k* persons at pixel *p* in the image.

(4) $$\begin{array}{c} L_{c}^{*}\left(p\right)=\frac{1}{n_{c}\left(p\right)}\sum_{k}L_{c,k}^{*}\left(p\right). \end{array}$$

**Figure 2:**
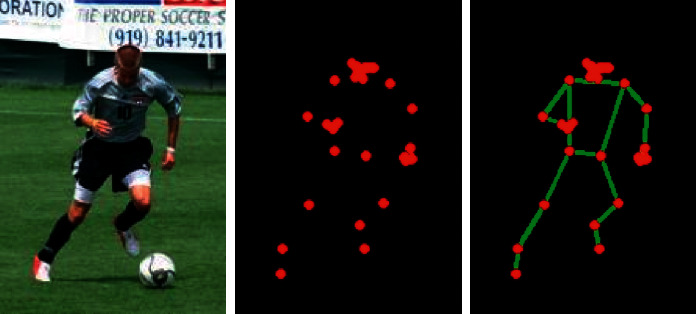
Human body posture design diagram.

Figure 2 is an image of the key nodes of the human body extracted by the above algorithm. The article extracts the key points of the image and then connects the key points to the image of the human body posture.

### 2.2. Generative Adversarial Network (GAN)

The generative adversarial network (GAN) was proposed by GOOdfellOw, using two convolutional neural networks using game training to generate samples similar to the original pictures [17–19].

**Figure 3:**
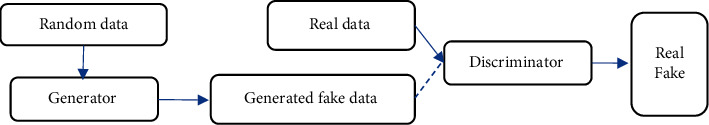
Generative adversarial network structure.

Figure 3 shows the structure of the generated confrontation network, which shows that GAN is primarily made up of a generating network *G* and a discriminating network D. It makes use of a conflicting relationship. The goal is for the generator to be able to generate data that is similar to real data distribution while giving the impression of being fake. GAN, which is aimed at the problem of data prediction, trains the generator to learn the data distribution and predicts the image distribution data. The success of confrontational ideas benefits the GAN network as well. Machine learning and artificial intelligence are two fields where the concept of confrontation has been introduced. Confrontation is present in the two behaviors of “game” and “competition.” Game machine learning is the result of combining game theory and machine learning.

The advantages and disadvantages of GAN: compared with other generative models, GAN has the following four advantages:

- From the actual results, GAN seems to produce better samples than other models.
- Most other frameworks require the generator network to have a certain functional form; for example, the output layer is Gaussian. It is important that all other frameworks require that the generator network be distributed with nonzero quality. The generative confrontation network framework can train any type of generator network and can learn to generate points only on thin manifolds close to the data.
- Any generator network and any discriminator will be useful because it does not need to design a model that follows any type decomposition.
- There is no need for reasoning during the learning process and no need to use Markov chain for repeated sampling (inference), thus avoiding difficult approximate calculation problems of probabilities.

### 2.3. The Generative Adversarial Network of Pose

**Figure 4:**
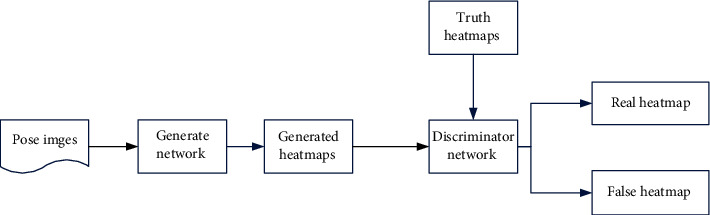
Human pose generation confrontation network structure model.

The model in this paper is divided into two networks, the generator and the discriminator, similar to the traditional GAN model [20, 21]. A convolutional network is the first network generator. The generator generates a set of heat maps that show the confidence score of each position of each key point after forward calculation. The second network discriminator uses the same architecture as the generator, but it encodes the input heat map along with the RGB image and decodes it into a new set of heat maps in order to distinguish between the real and fake heat maps. The generator is strengthened to generate the human body posture as a result of the game between the generator and the discriminator, and the discriminator's human joints are gradually improved. The existing pose estimation model is improved through continuous training, as shown in Figure 4.

#### 2.3.1. Generator

The generator is responsible for predicting the posture, and its role is to generate accurate information about the key points of the human body. Of course, as part of the generational confrontation, the main function of the generator is to allow the generated key points to fool the final discriminator, so that the discriminator cannot distinguish whether the current key point heat map is actual or generated by the generator.

That is to say, the purpose of the counter loss is to make the key points generated by the generator conform to a more reasonable posture. More directly, the purpose of *L*_adv_ is to make the false heat map generated by the generator fool the discriminator as much as possible, making it impossible to distinguish between the GT heat map and the false heat map. The process of generating confrontation is reflected here. Finally, the loss shown in formula 3 is used to optimize the generator. *λ* is a hyperparameter. The generator loss function is as follows:

(5) $$\begin{array}{c} L_{\text{MSE}}=\sum_{i=1}^{N}\sum_{j=1}^{M}{\left(C_{ij}-{\hat{C}}_{ij}\right)}^{2},\\ L_{\text{adv}}=\sum_{i=1}^{N}{\left({\hat{C}}_{j}-D\left({\hat{C}}_{j},X\right)\right)}^{2},\\ L_{G}=L_{\text{MSE}}+\lambda L_{\text{adv}}, \end{array}$$

where *L*_MSE_ is the loss of the generator, *L*_adv_ is the counter loss from the resolver, and *λ* is a hyperparameter.

#### 2.3.2. Differentiator

The discriminator's goal is to improve human joints over time. The goal is to determine whether the input heat map is the real thing or a forgery created by the generator. The discriminator's ultimate training goal is to be able to tell the difference between the data generated by the generator and the real heat map as much as possible. As a result, a confrontation game with the generator is formed.

The encoded new heat map is obtained by inputting the false heat map generated by the generator into the discriminator. And calculate the distance between the new heat map and the fake heat map generated by the generator to calculate the *L*_fake_ loss. As mentioned above, the purpose of the discriminator is to distinguish the false heat map from the actual heat map as much as possible. That is to say, the classifier hopes that the output reconstructed heat map after the actual heat map input is as close as possible to the actual one. The loss function of the discriminator is as follows:

(6) $$\begin{array}{c} L_{\text{real}}=\sum_{j=1}^{N}{\left(C_{j}-D\left(C_{j},X\right)\right)}^{2},\\ L_{\text{fake}}=\sum_{j=1}^{N}{\left({\hat{C}}_{j}-D\left({\hat{C}}_{j},X\right)\right)}^{2},\\ L_{G}=L_{\text{real}}+k_{t}L_{\text{fake}},\\ k_{t+1}=k_{t}+\lambda_{k}\left(L_{\text{real}}-L_{\text{fake}}\right). \end{array}$$

The *kt* in the above formula is used to constrain the capability of the discriminator. Constraining *kt* through the formula can make the network easier to train. But GAN is unstable and difficult to train because the discriminator converges too fast. This will cause the network to easily collapse and train an invalid generator. In response to this problem, in order for the network to be able to generate enough truth to deceive the discriminator. By studying the relationship between *L*_fake_ and *L*_real_, it can be found that, with the increase of kt, *L*_fake_ has more advantages, which makes the discriminator more training to recognize the generated heat map. But *kt* is not an infinite stack. To this end, this paper extracts the confidence heat map information from the discriminator and feeds it back into the generator, thereby enhancing the generator's accurate prediction of the key nodes of the human body on the ground.

### 2.4. Confidence Evaluation Mechanism

The generator can pay more attention to prominent body joints because the confidence evaluation mechanism uses normalization technology. As a result, the algorithm's body node's detection accuracy increases. To determine the key points' credibility, compare the generated heat map with the actual heat map. Following that, improve the algorithm's key point extraction accuracy. The following are the techniques.

The discriminator is used to process the generated heat maps and truth heat maps in this paper—confidence heat maps that were derived from the data. The pose resolver requires two tags to calculate two losses. The identification network marks the real samples as 1 and the pseudosamples as 0, in the traditional work of image conversion using GAN. We discovered that using only 0 or 1 as the label for the human body pose estimation problem makes convergent network difficult. As a result, this paper relies on the discriminator's confidence heat maps to direct the generator's data generation.

Set the initial setting of the generator to a low-confidence heat map. Then the confidence discriminator classifies the result as fake. The generator will self-optimize based on this result error to generate a higher degree of confidence until it can make the discriminator think that the result of the generator is true. Therefore, even if there is occlusion, this process will help the generator to generate a high-confidence heat map.

The vector corresponding to the feature map with the corresponding key point value is 1, and the vector corresponding to the feature map without the key point is 0, where *di* is the normalized distance between the predicted position of the *i-*th body part and the true position. For fake samples, if the predicted body part is far away from the real position, then the posture in this image is obviously not believable to the body structure. Therefore, if the distance is relatively close, the corresponding vector is 1; otherwise the corresponding vector is 0.

Then the corresponding value of C_fake_ is shown in the following formula. When the generated key point heat map is relatively close to the actual heat map (the distance is less than the threshold), the corresponding vector is 1. Otherwise, the corresponding vector is 0.

(7) $$\begin{array}{c} C_{\text{fake}}=\left\{\begin{array}{cc} v, & \text{if }\left|\left|d_{i}-{\hat{d}}_{i}\right|\right|<\tau ,\\ 0, & \text{if }\left|\left|d_{i}-{\hat{d}}_{i}\right|\right|\geq \tau . \end{array}\right) \end{array}$$

Among them, for the feature map, first transform *x* into feature space *f* and *g*. And make it satisfy

(8) $$\begin{array}{c} f\left(x\right)=W_{f}\left(x\right),\\ g\left(x\right)=W_{g}\left(x\right). \end{array}$$

*W*_*f*_(*x*) is the weight matrix of generated heat maps. *W*_*g*_(*x*) is the weight matrix of confidence heat maps.

The distance is normalized to [0, 1] through element transformation.

(9) $$\begin{array}{c} d_{i}=\frac{e^{{\left\|f\left(x_{i}\right)-g\left(x_{i}\right)\right\|}_{2}}-1}{e^{{\left\|f\left(x_{i}\right)-g\left(x_{i}\right)\right\|}_{2}}+1}. \end{array}$$

**Figure 5:**
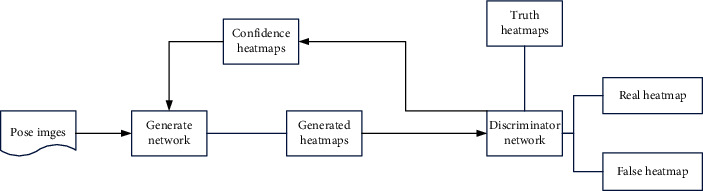
Introduces confidence in human posture generation of adversarial network structures.

In the end, the best key points are obtained, and the network model of the algorithm in this paper is shown in Figure 5.

**Figure 6:**
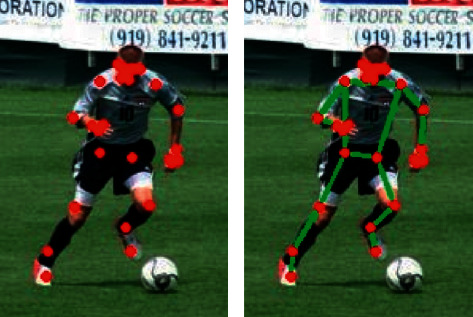
Human posture setting effect.

Figure 6 is the result of adopting the human body pose generation confrontation network structure that introduces confidence. The human body posture is generated by the generator, and the discriminator is gradually improving the human joints. Finally, the normalization technology is used to perform confidence processing on the generator's generated data to retain the correct key nodes. In turn, the detection accuracy of the body node of the algorithm is improved. The effect is shown below.

Through observation, it can be seen that the above method can accurately locate the characters of the image and can accurately locate all key nodes of the whole body. In order to better verify the feasibility of the algorithm, the following is a detailed experiment.

## 3. Simulation Experiments

### 3.1. Experimental Environment and Datasets

The experimental environment of this article: Ubuntu 16.04.4 platform is implemented by Python programming with open CV 3.4 and Tensorflow 1.5 frameworks, processors: Intel Core i7 7700 HQ (up-to 3.8 GHz), 16 GB Memory, nVidia Geforce GTX 1060 6 GB VGA, training with a total of 20,000 images, training time of 80 hours.

Dataset: this paper selects three widely used human posture datasets as follows.

#### 3.1.1. LSP Human Posture Estimation Dataset (Address: https://sam.johnson.io/research/lsp.html)

Leeds Sports Pose is a dataset of sports postures divided into categories such as athletics, badminton, baseball, gymnastics, parkour, soccer, volleyball, and tennis, all of which are images from physical activity and thus very challenging in terms of appearance, particularly joints. The dataset consists of 2,000 annotated images of postures primarily sourced from Flickr and labeled with the labels shown above. The length of the most prominent person has been scaled to approximately 150 pixels in these images. Each image has 14 joint positions labeled.

#### 3.1.2. FLIC Human Detection Dataset (Address: https://bensapp.github.io/flic-dataset.html)

FLIC is a dataset of images that label people in frames of movies, which contains 5,003 images collected from mainstream Hollywood movies. The training images are derived from a character detector running in 30 movies and are manually annotated for them after the pictures are taken, including 10 upper body joints. In addition, there are 5 median markers in the image to ensure that the outlier annotation is robust. Publishers voluntarily reject images that are obscured or have too low picture clarity and reserve 20% of about 1016 images for testing.

#### 3.1.3. MPII Human Posture Estimation Dataset (Address: https://human-pose.mpi-inf.mpg.de/)

MPII is a dataset for evaluating human posture estimates and related benchmarks, with approximately 25,000 images and more than 40,000 people with annotated joints, systematically collecting images using established taxonomies of human activity.

Overall, the dataset covers 410 human behaviors and each image provides an activity tag, each image is from a YouTube video, and provides a related unnoticed framework. In addition, the test set's annotations include body part occlusion, 3D torso, and head orientation.

### 3.2. Evaluate Metrics

The article selects the LSP human pose estimation data set for verification and uses the percentage of correct key points (PCK) to measure the performance of the LSP data set. We choose the traditional OpenPose algorithm; [20] algorithm, and [21] algorithm to verify the accuracy of the algorithm by calculating the percentage of correct detections that fall within the normalized distance and recording the data results.  Table 1 shows the data obtained by measuring the key nodes of the whole body using the abovementioned algorithms, as shown in the table.

**Figure 7:**
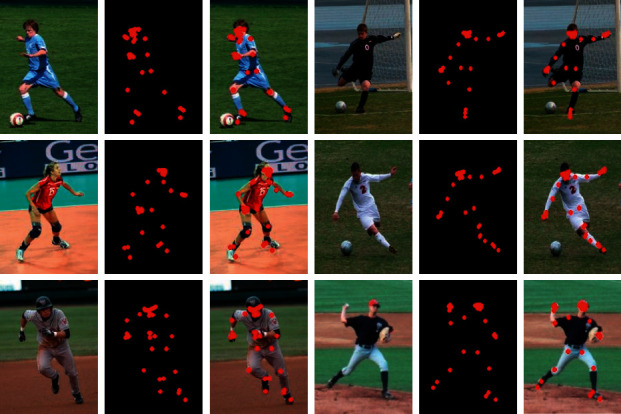
Key point extraction rendering.

This article selects head, shoulder, elbow, wrist, hip, knee, and ankle as 7 nodes for data comparison; through the observation of the data, it can be seen that the proposed algorithm synthesis data is better than several other algorithms. Among them, the algorithm of this paper has achieved the best results in head, shoulder, elbow, and ankle of 96.64%, 95.16%, 95.97%, and 95.16%, respectively. The average values of each algorithm were 89.83%, 92.30%, 94.71%, and 95.37%, respectively; you can see that using the algorithm of this article in the key point extraction effect is the best. We aimed to verify whether the algorithm can still maintain good results in different environments. In this paper, we use LSP human pose estimation data set to verify that the difference is motion pictures. After testing, the result of the test is shown in Figure 7.

Through observation, it can be seen that the data obtained by the algorithm of this paper can accurately locate the characters in the image. And in the key extraction renderings of the human body, the key points will not appear to be missing and elegant. The algorithm can accurately locate according to the different actions of the characters. It further verifies the key point extraction accuracy of the algorithm.

**Figure 8:**
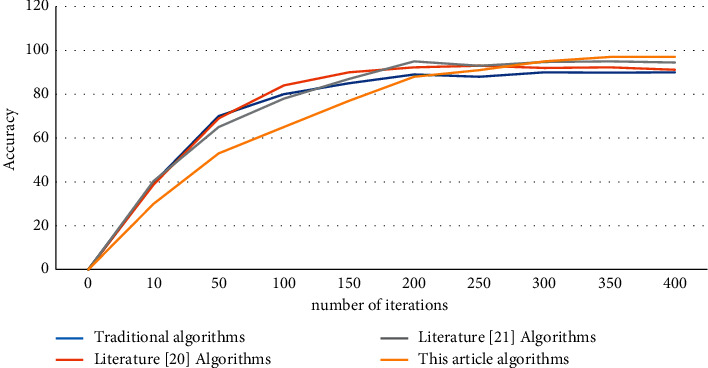
The number of iterations and the accuracy of each algorithm into the data curve.

By collecting the iterative data of each algorithm, and according to the number of iterations of the algorithm, the data curve shown in Figure 8 can be obtained, which is marked according to the number of iterations and accuracy of each algorithm, as shown in Figure 8.

By observing the curve data graph in Figure 8, it can be seen that the final accuracy of the algorithm in this paper is higher than the other three algorithms, but the cost is that the number of iterations required by the algorithm is higher than that of other algorithms. It can be seen that the accuracy of the algorithm in this paper is stable at about 96.20%, and the number of iterations needs to meet more than 350 times to achieve. In contrast, the number of iterations of other algorithms can reach the highest value of the algorithm at about 200 times.

### 3.3. Qualitative Analysis

**Figure 9:**
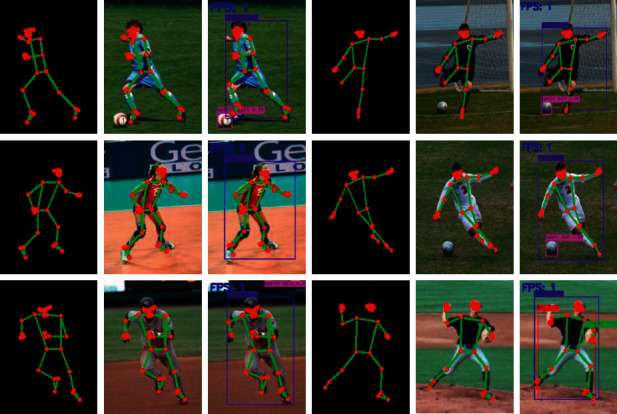
Human body posture design effect figure.

The article selects the LSP human pose estimation data set for qualitative analysis. And on the basis of the above, the key points are connected and displayed in the actual image. At the same time, combined with target detection, on the basis of human posture, the detection effect of target detection is strengthened. Reflect in the following. The detection image is shown in Figure 9.

By observing Figure 9, it can be seen that using the algorithm in this paper, good key point extraction and target locking can be maintained in different motion environments. This method strengthens the processing of extraction and confidence through high-performance network architecture and loss function. In turn, the high-precision human body posture positioning effect is obtained, and the pedestrian detection capability is better.

### 3.4. Comparison of Average Running Time

In this paper, we train on the MPII human body pose estimation data set, LSP human body pose estimation data set, and FLIC human body detection data set. And the various running times are compared, and the results are shown in Table 2.

Verify the algorithm's running time in various environments by observing the data differences to see if the algorithm can maintain a stable running effect. We can see that the algorithm in this paper performs well in each data set by comparing the data in Table 2. The model used in this paper is the largest of them all, but in terms of running time, it can be applied to a variety of other algorithms. The effect expressed in the LSP data set is the best, with a time of only 0.229 s, effectively proving the algorithm's feasibility.

## 4. Conclusion

The article proposes an adaptive generative adversarial network to improve human pose detection algorithm. In the algorithm model of this article, the generator is responsible for predicting the posture, and the discriminator is for the gradual improvement of the human joints. However, in the actual training process, the predicted nodes generated by the generator sometimes deviate from reality. Therefore, this paper introduces a confidence evaluation mechanism into the system to determine the credibility of the generated key points, so that the generator can pay more attention to the prominent body joints, thereby improving the detection accuracy of the algorithm. Finally, by using three standard data sets to evaluate the algorithm, it can be seen that the algorithm is superior to other algorithms in terms of detection accuracy and running time. This effectively proves the effectiveness of our method. Compared with the existing methods, the algorithm in this paper can use the GAN model to search for the positions of known persons and search for poses in these areas and improve the prediction of subsequent poses based on the existing data, and the accuracy is greatly improved. In the next step, we will conduct in-depth research on the improvement of model training stability and how to improve the resolution of generated images.

## Figures and Tables

**Table 1:** Correct detection percentage.

Method	Head	Shoulder	Elbow	Wrist	Hip	Knee	Ankle	Average value
Traditional algorithms	89.01	85.40	91.72	89.06	89.90	91.45	92.30	89.83
Literature [20] algorithms	91.96	91.57	88.32	90.21	94.65	94.90	94.51	92.30
Literature [21] algorithms	93.81	98.76	93.57	94.51	95.68	93.05	93.56	94.71
This article algorithm	96.64	95.16	95.97	94.73	94.88	95.08	95.16	95.37

**Table 2:** Average running time under each data set.

Method	Model size (M)	Runtime(s) on MPII test set	Runtime(s) LSP test set	Runtime(s) FLIC test set
Traditional algorithms	162.1	1.242	0.234	1.07
Literature [20] algorithms	157.3	1.201	0.325	0.921
Literature [21] algorithms	196.21	1.266	0.238	0.951
This article algorithm	201.2	1.191	0.229	0.806

## Data Availability

The data used to support the results of this study need to be obtained with the consent of the corresponding author.
